# Exercise as an Alternative Approach for Treating Smartphone Addiction: A Systematic Review and Meta-Analysis of Random Controlled Trials

**DOI:** 10.3390/ijerph16203912

**Published:** 2019-10-15

**Authors:** Shijie Liu, Tao Xiao, Lin Yang, Paul D. Loprinzi

**Affiliations:** 1 School of Physical Education and Sport Training, Shanghai University of Sport, Shanghai 200438, China; 1911111017@sus.edu.cn; 2 College of Mathematics and Statistics, Shenzhen University, Shenzhen 518060, China; taoxiao@szu.edu.cn; 3 Cancer Epidemiology and Prevention Research, Alberta Health Services, Calgary, AB T2S 3G3, Canada; lin.yang@ahs.ca; 4 Departments of Oncology and Community Health Sciences, Cunning School of Medicine, University of Calgary, Calgary, AB T2N 4N1, Canada; 5 Exercise & Memory Laboratory, Department of Health, Exercise Science and Recreation Management, The University of Mississippi, Oxford, MS 38677, USA

**Keywords:** exercise, Tai chi, smartphone addiction, internet addiction

## Abstract

Background: With the emergence of electronic products, smartphones have become an indispensable tool in our daily life. On the other hand, smartphone addiction has become a public health issue. To help reduce smartphone addiction, cost-effective interventions such as exercise are encouraged. Purpose: We therefore performed a systematic review and meta-analysis evaluating existing literature on the rehabilitative effects of exercise interventions for individuals with a smartphone addiction. Methods: We searched PubMed, Web of Science, Scopus, CNKI, and Wanfang from inception to September 2019. Nine eligible randomized controlled trials (RCT) were finally included for meta-analysis (SMD represents the magnitude of effect of exercise) and their methodological quality were assessed using the PEDro scale. Results: We found significant positive effects of exercise interventions (Taichi, basketball, badminton, dance, run, and bicycle) on reducing the total score (SMD = −1.30, 95% CI −1.53 to −1.07, *p* < 0.005, *I*^2^ = 62%) of smartphone addiction level and its four subscales (withdrawal symptom: *SMD* = −1.40, 95% CI −1.73 to −1.07, *p* < 0.001, *I*^2^ = 81%; highlight behavior: SMD = −1.95, 95% CI −2.99 to −1.66, *p* < 0.001, *I*^2^ = 79%; social comfort: SMD = −0.99, 95% CI −1.18 to −0.81, *p* = 0.27, *I*^2^ = 21%; mood change: SMD = −0.50, 95% CI 0.31 to 0.69, *p* = 0.25, *I*^2^ = 25%). Furthermore, we found that individuals with severe addiction level (SMD = −1.19, *I*^2^ = 0%, 95%CI:−1.19 to −0.98) benefited more from exercise engagement, as compared to those with mild to moderate addiction levels (SMD = − 0.98, *I*^2^ = 50%, 95%CI:−1.31 to −0.66); individuals with smartphone addiction who participated in exercise programs of 12 weeks and above showed significantly greater reduction on the total score (SMD = −1.70, *I*^2^ = 31.2%, 95% CI −2.04 to −1.36, *p* = 0.03), as compared to those who participated in less than 12 weeks of exercise intervention (SMD = −1.18, *I*^2^ = 0%, 95% CI−1.35 to −1.02, *p* < 0.00001). In addition, individuals with smartphone addiction who participated in exercise of closed motor skills showed significantly greater reduction on the total score (SMD = −1.22, *I*^2^ = 0 %, 95% CI −1.41 to −1.02, *p* = 0.56), as compared to those who participated in exercise of open motor skills (SMD = −1.17, *I*^2^ = 44%, 95% CI−1.47 to −0.0.87, *p* = 0.03). Conclusions: Exercise interventions may have positive effects on treating smartphone addiction and longer intervention durations may produce greater intervention effects.

## 1. Introduction

With the emergence of electronic products, smartphones are a type of electronic device that is nowadays widely used in our daily life [1,2]. The China Internet Network Information Center has reported that smartphone users have reached around 788 million in 2018, of which 20% were student users, and users of this age group are likely to continue to increase [3]. It was reported that 20% of teenagers in Spain have a smartphone addiction, and this proportion is up to 36% in the UK [4]. Worldwide, Chinese teenagers are second only to Brazil in terms of smartphone addiction [5]. As a new social phenomenon, smartphone addiction is divided into behavioral and psychological characteristics. Excessive uses of smartphones may cause severe distraction. Typical behavior is looking at a phone every few minutes or once in a while to determine whether there is a new message. Present studies provided strong evidence of using smartphone-based app accounts for a large proportion of typical daily smartphone usage in smartphone addiction [6]. Games and information, health and entertainment program apps are more addictive among participants [6,7].

Smartphone addiction is defined in terms of criteria of behavioral addiction, including saliency, impulsivity, highlight behavior, and withdrawal symptoms. Individuals with a smartphone addiction lack control over smartphone use [8], thereby interfering with other activities [9]. Smartphone addiction is different from smartphone dependency; the former emphasizes withdrawal and impulsivity, while the latter emphasizes implicit behaviors. Currently, measurement of smartphone addiction [10,11,12] is based on Yong’s [13] Internet Addiction Text and Goldberg’s [14] diagnostic criteria of internet addiction. Undoubtedly, a smartphone provides great conveniences for work, study, and social activities, but excessive use of smartphones have been shown to associate with physical discomfort (e.g., neck pain and stiffness, dry eyes, and headaches) [15], psychological symptoms (e.g., increased loneliness, reduced self-esteem, reduced productivity, loss of attention, sleep problem, depression and anxiety) [16,17], and increased risk of accidents (traffic accidents, falling/slipping, and bumps/collisions) [18]. Smartphone addiction-induced symptoms may reduce the social abilities and quality of life. Therefore, smartphone addictions have increasingly become a public health issue.

Currently, static behavior in front of a screen is a hot topic in fields of international physical activity and public health [19]. Specifically, a high frequency use of smartphone and internet is considered as sedentary [20,21], which is related to higher body mass index and lower level of physical activity [22]. Long-term sedentary behavior with low energy consumption may lead to obesity or metabolic syndrome. In addition, previous research suggests that the intensity of smartphone use is adversely correlated with intensity of physical activities, and that smartphone addiction can be effectively alleviated by systematic physical activities (mainly, by behavioral therapies and adjuvant therapies) [22]. While behavioral therapies (cognitive behavioral therapy, reality therapy, family-based group therapy and positive psychological intervention) have been widely recognized as an effective way to reduce Internet/smartphone addiction, adjuvant therapies (exercise or physical activity) have attracted research attention as well [22]. Previous research shows that exercise interventions can restore and adjust highly exciting nerve cells and enhance their adaptability to external changes [23,24]. Zhu [25] and Zhang [26] found that exercise can reduce smartphone addiction among teenagers, but Zhou [27] pointed out that such effects exist but are limited. Although with inconsistent results to some extent, these studies suggest that exercise interventions are beneficial and should be considered as an alternative non-pharmacological approach for treating individuals with smartphone addiction [28,29,30,31,32,33].

With the number of these experimental studies increasing, a systematic/narrative review on this topic was conducted and published in 2013 [22], but this review did not draw a firm conclusion. This previous review, however, employed a qualitative review approach, as opposed to a quantitative meta-analytic approach. Under this condition, we performed a comprehensive search and objectively evaluated the existing evidence. Results of this quantitative review provide implications for health professionals and researchers.

## 2. Methods

### 2.1. Data Sources

To retrieve potential articles, we searched PubMed, Web of Science, Scopus, CNKI, and Wanfang, from inception until September, 2019. Two panelists independently searched the Chinese/English published articles, and used the following two groups of search terms: (1) mobile phone dependence, mobile addiction, smart phone dependence, or smart phone addiction; and (2) exercise, sports, motor skills, physical education, football, basketball, mind-body exercise, Tai Chi, yoga, badminton, volleyball, or outdoor games. If the article was incomplete or not available, we contacted the corresponding author of the primary studies via email.

### 2.2. Criteria for Study Selection 

Studies were considered eligible if they met the inclusion criteria as follow: (1) participants, who were college students and identified as mobile/smart phone dependent/addicted in the primary studies; (2) the experimental group used exercise as the main intervention; (3) they were randomized controlled trials (RCT); (4) employed a pre/post design; (5) a minimum of one outcome was reported (all outcome measures are listed in Section 2.3). Exclusionary criteria: (1) descriptive study or case-control study; (2) no control group; or (3) incomplete data. Two researchers independently screened the initially retrieved documents against the aforementioned inclusion criteria. When disagreements between the two independent researchers occurred, a third reviewer evaluated the original study to reach a consensus.

### 2.3. Data Extraction and Synthesis

Two independent researchers (S.J.L. and L.Y.) extracted the information of first author of each primary study, year of publication, place of implementing experimental procedures, participants’ features (the number of participants and its attrition rate, age of participants, and smartphone addiction level), details of intervention program (dosage and duration), outcome measures, and occurrence of adverse event and follow-up period. If there was any disagreement or the information was inconsistent, it was discussed with a third reviewer (T.X.).

The outcome measures of these primary studies are as follow: (1) smartphone addiction tendency scale (MPATS): including 17 items and four sub-scales (withdrawal symptoms, highlight behavior, social comfort, mood change), 0 to 80 scores, higher scores mean more serious mobile phone addiction tendency; (2) smartphone addiction index scale (MPAI), including 17 items and two sub-scales (withdraw, uncontrolled, escaping, inefficiency), 0 to 85 scores, higher scores show more serious mobile phone addiction tendency; (3) smartphone addiction scale for college students (SAS-C), including five dimension (persistence, conflict, withdraw, technical, salience ) and 20 items, higher scores mean more dependent.

According on DSM-IV, we unified the similarity dimensions of the above three scales as follows: withdrawal symptoms (negative emotional reactions when someone cannot use smartphone), highlight behavior (the importance for using mobile phone), conflict (the negative effect of mobile phone on individuals), and social comfort (the effect of using mobile phone in social interaction). Because both uncontrolled and salience are emphasizing the importance of using mobile phone on individuals, we merged them into one item of highlight behavior [34].

### 2.4. Methodological Quality Assessment for Included Studies

Two independent researchers (S.J.L. and L.Y.) performed the methodological quality assessment for the included studies according to the Physiotherapy Evidence Database scale, or PEDro scale. The PEDro scale is designed for the use of best evidence to enhance the effectiveness of physical therapy services. Its assessment includes 11 items, with higher scores representing better methodological quality of the corresponding study [35]. A third researcher was consulted only if the previous two researchers had a disagreement on any items of the PEDro scale. Given that it is unrealistic to blind participants and instructors to an exercise intervention, these two items were removed from the original 11 PEDro scale items. Thus, the scale used in the current review consisted only of nine items: eligibility criteria, randomization, concealed allocation, blinded of assessor, point estimates, baseline equivalence, between-group comparison, a retention rate of 85% and above, and outcome measured integrity.

### 2.5. Meta-analysis for Outcomes 

This meta-analytic method was evaluated via software from Comprehensive Meta-Analysis Software (Bio. Stat. Inc., Englewood, NJ, USA). In the meta-analysis of each outcome, we selected standardized mean difference (SMD as pooled effect size reflects magnitude of effect of exercise intervention), with its 95% confidence interval (CI). The heterogeneity of included studies was tested using Q statistic for homogeneity test with a 0.1 significance level; the heterogeneity statistic I-squared was reported (25%, 50%, and 75% cutoffs represent small heterogeneity, medium heterogeneity, and large heterogeneity, respectively) [36]. We used the Funnel plot to visually observe whether publication bias existed. If scatter was symmetrically distributed on both sides of the funnel, it indicated that the result had no publication bias, otherwise, the publication bias likely existed.

### 2.6. Assessment of Certainty of Evidence for Meta-analysis Results

We used GRADE (grading recommendations assessment development and evaluation) [37] to classify the overall certainty of evidence of the significant effect on the outcome (*p*-value in random effect model < 0.05), using four possible levels:

I (high): the true effect is similar to the estimated effect with confidence;
II (moderate): the true effect is most likely close to the estimated effect;
III (low): the true effect might be markedly different from the estimated effect;
IV (very low): the true effect is most likely markedly different from the estimated effect.

Assessment criterion for the GRADE are based on the following five assessment items:

1. Risk of bias: present if the risk of bias of included studies in meta-analysis is large in our judgement;
2. Imprecision: present if sum of sample sizes of all individual studies included in meta-analysis is less than 500;
3. Inconsistency: present if heterogeneity statistic I-squared in meta-analysis is greater than 50%;
4. Indirectness: present if the intervention studied in meta-analysis is not directly relevant to the outcome;
5. Publication bias: present if the p-value of Begg’s test for the publication bias in meta-analysis is less than 0.05.

All meta analyses we conducted were based on randomized controlled trials, evidence certainty starting at level I. The presence of any of these items would rate the evidence certainty down with one level until it reached level IV.

## 3. Results

### 3.1. Study Selection

**Figure 1:**
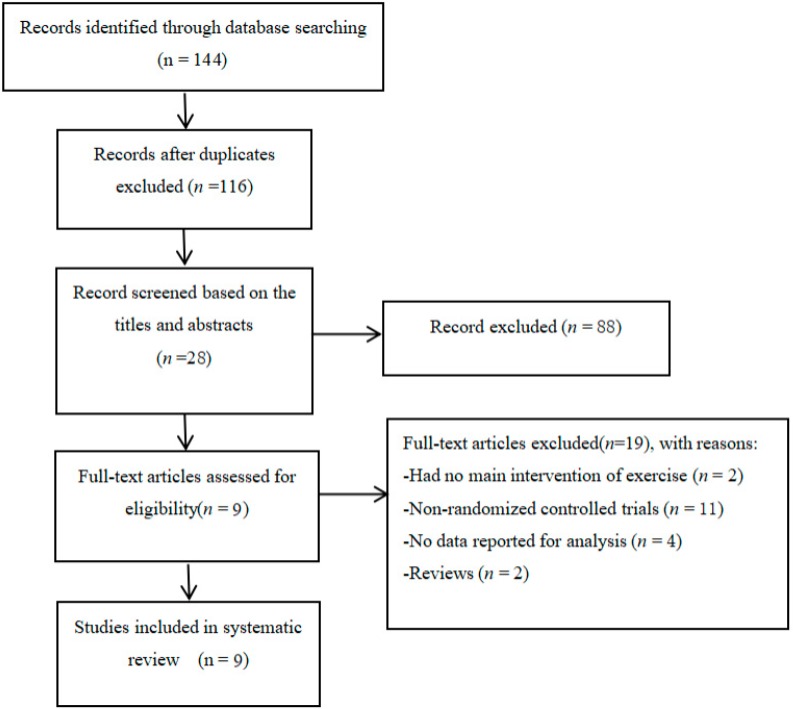
Flow of study selection.

Figure 1 describes the detailed search process of our meta-analysis. A total of 144 potential studies were searched and 28 full-text publications were screened for further evaluation. After eliminating the irrelevant studies (*n* = 19), nine studies were identified for data extraction and quality assessment.

### 3.2. Characteristics of Eligible Studies

Table 1 shows characteristics of the 9 included studies. Nine RCTs were conducted in different provinces of China and published between 2015 and 2019, and the included studies employ a pre/post design. A total of 1582 undergraduates (exercise = 792 and control = 790) were included. Participants were 20.43 years of age (SD = 1.52 years), two studies [14,15] selected only freshmen. In term of interventions, four studies [25,26,31,33] used single intervention independence, other studies [27,28,29,30,32] used mixed exercise intervention. Intervention duration ranged 8–12 weeks with 3–5 sessions weekly, with each session lasting 45–120 minutes. Additionally, behavioral outcomes of these nine RCTs were measured by the Smartphone Addiction Tendency Scale (MAPTS) (*n* = 5), the Smartphone Addiction Scale for College Students (SAS-C) (n = 1) or smartphone addiction index (MPAI) (*n* = 3). During the intervention period, no adverse events occurred. Only one of included studies used follow-up assessment.

### 3.3. Methodological Quality Assessment

We presented information on the methodological quality assessment in Table 2. All nine studies were RCTs, but the appropriate methods such as concealed allocation methods and blinded of assessor were not guaranteed. Blinding of assessors were employed in two studies [28,31]. The quality scores of the eligible RCTs were 6 to 8 points.

### 3.4. Data Synthesis of Behavioral Outcomes

**Figure 2:**
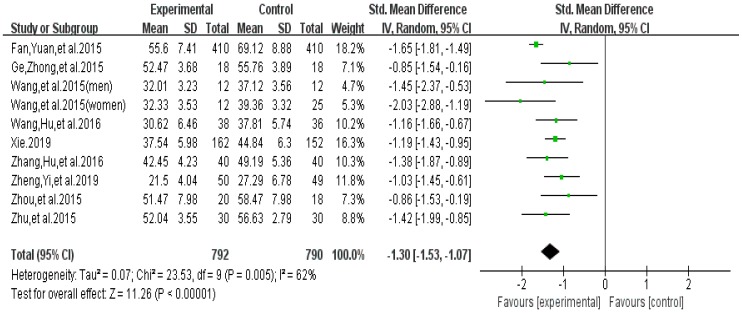
Effect of exercises on total score of smartphone addiction.

**Figure 3:**
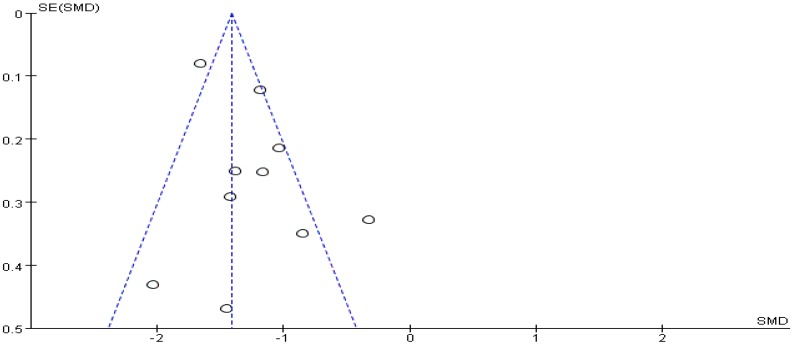
Funnel plot for the publication bias of the total score of smartphone addiction.

Meta-analysis focused on both total score of addiction level and four individual sub scores; we list the synthesized results for the effects of exercises versus control condition systematically (Table 3). In the meta-analysis of nine studies (10 pairwise comparisons, *n* = 1582), exercise interventions showed significant effects on reducing addiction level/the total score (SMD = − 1.30, 95% CI −1.53 to −1.07, *p* = 0.005, *I*^2^ = 62%; Figure 2), in comparison to control groups; funnel plot showed that the distribution of the included studies was symmetric (Figure 3), and the Begg test result was *p* = 0.377, indicating no evidence of publication bias in the included studies. 

#### 3.4.1. Effect of Exercise Interventions on Withdrawal Symptoms

**Figure 4:**
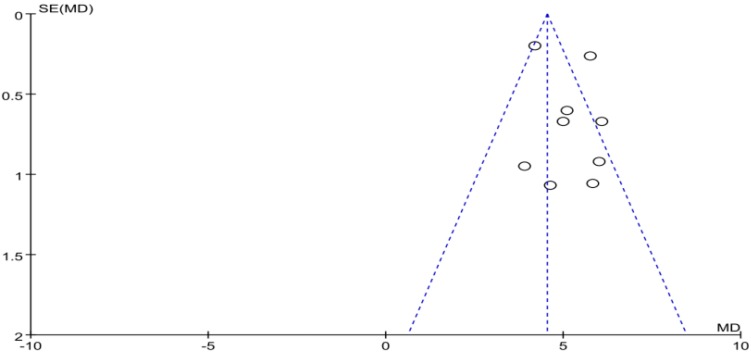
Funnel plot for publication bias of withdrawal symptoms.

**Figure 5:**
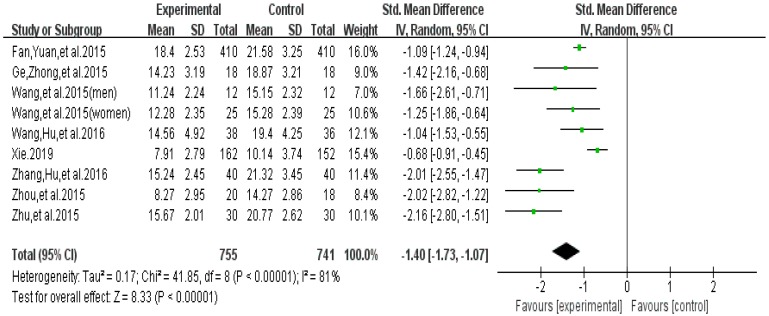
Effect of exercises on withdrawal symptoms.

The funnel plot of withdrawal symptoms is displayed in Figure 4. Funnel plot showed that the distribution of the included studies was symmetric, and the Begg test result was *p* = 0.653, indicating no evidence of publication bias in the included studies. In the meta-analysis of eight studies (nine pairwise comparisons, *n* = 1496) exercise showed a significant effect on improving withdrawal symptoms of college students with smartphone addiction (SMD = −1.40, 95% CI −1.73 to −1.07, *p* < 0.001, *I*^2^ = 81%, Figure 5).

**Figure 6:**
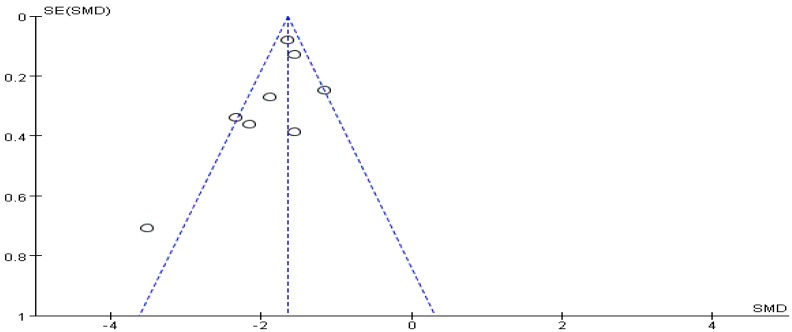
Funnel plot for publication bias of highlight behavior.

**Figure 7:**
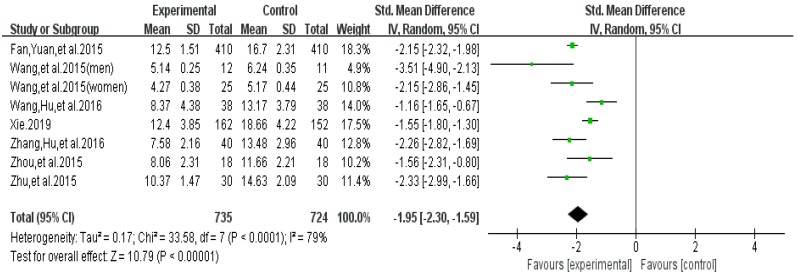
Effect of exercises on highlight behavior.

The funnel plot of highlight behavior outcome is displayed in Figure 6. The funnel plot showed that the distribution of the included studies was symmetric, and the Begg test result was *p* = 0.482, indicating no evidence of publication bias in the included studies. A meta-analysis of the remaining seven RCTs (eight pairwise comparison, *n* = 1459) showed positive effects of exercise for improving the highlight behavior of college students with smartphone addiction (SMD = −1.95, 95% CI −2.99 to −1.66, *p* < 0.001, *I*^2^ = 79%; Figure 7).

**Figure 8:**
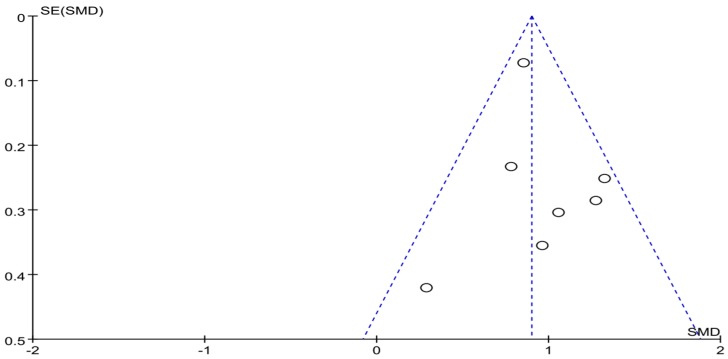
Funnel plot for publication bias of social comfort.

**Figure 9:**
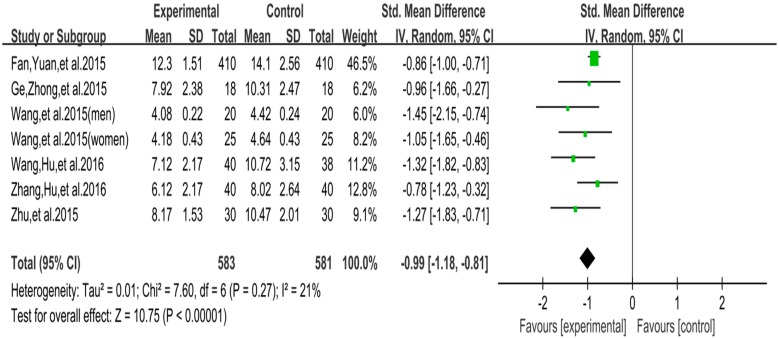
Effect of exercises on social comfort.

The funnel plot of social comfort is displayed in Figure 8. Funnel plot showed that the distribution of the included studies was symmetric, and the Begg test result was *p* = 0.955, indicating no evidence of publication bias in the included studies. In the meta-analysis of six studies (seven pairwise comparison, *n* = 1164), exercise showed significant effects on improving social comfort of college students with smartphone addiction (SMD = −0.99, 95% CI −1.18 to −0.81, *p* = 0.27, *I*^2^ = 21%; Figure 9).

**Figure 10:**
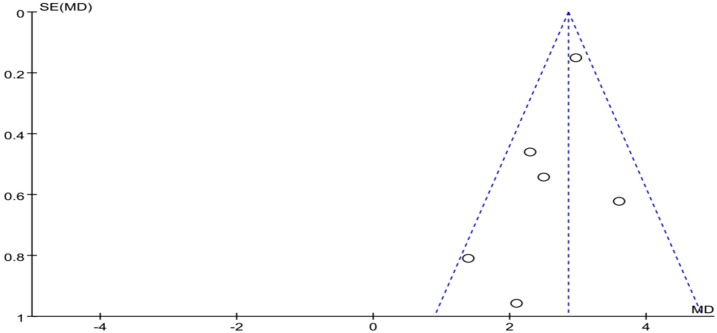
Funnel plot for publication bias of mood change.

**Figure 11:**
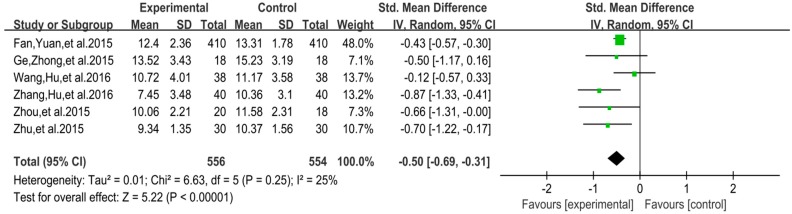
Effect of exercises on mood change.

The funnel plot of mood change is displayed in Figure 10. Funnel plot showed that the distribution of the included studies was symmetric, and the Begg test result was *p* = 0.891, indicating no evidence of publication bias in the included studies. In the meta-analysis of six studies (*n* = 1110), exercise showed a significant effect on improving mood change of college students with smartphone addiction (SMD = −0.50, 95% CI 0.31 to 0.69, *p* = 0.25, *I*^2^ = 25%; Figure 11).

#### 3.4.2. Summary of Certainty of Evidence

Assessment of the certainty of evidence for the highly significant effects of exercises on total score of addiction level and four individual sub scores by the GRADE (grading recommendations assessment development and evaluation) criterion is given in Table 4.

#### 3.4.3. Subgroup Analysis for Total Score of Smartphone Addiction

**Figure 12:**
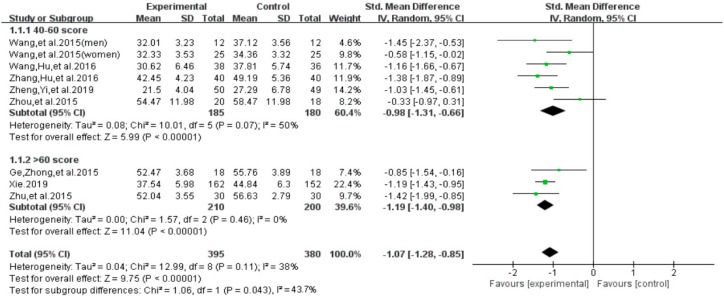
Effects of exercises on level of smartphone addiction.

For the total score of smartphone addiction, we performed subgroup analyses on baseline dependence level (40 to 60 points = mild-to-moderate level versus more than 60 points = severe level) [22,23,24] and intervention duration (less than 12 weeks versus 12 weeks and above) (Figure 12). With regard to the baseline dependence level, we found a significant group difference on the total score of smartphone addiction (SMD = −1.07, 95% CI −1.28 to −0.85, *p* = 0.043). Furthermore, results indicated that individuals with a severe-level of smartphone addiction at baseline significantly benefited more (SMD = −1.19, *I*^2^= 0%, 95% CI: −1.19 to −0.98), when compared to those with the mild-to-moderate level of smartphone addiction (SMD = − 0.98, *I*^2^ = 50%, 95% CI: −1.31 to −0.66).

**Figure 13:**
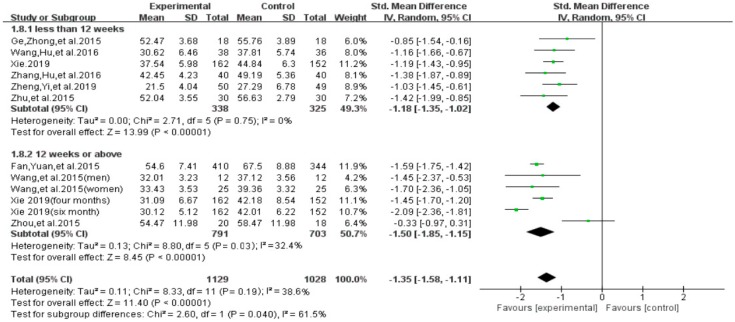
Effect of intervention duration on smartphone addiction patients.

With regard to the intervention duration, results of subgroup analysis indicated a significant group difference on the total score of smartphone addiction (SMD = −1.42, 95% CI −1.68 to −1.17, *p* = 0.021, Figure 13). Furthermore, results indicated that individuals with smartphone addiction who participated in exercise intervention of 12 weeks and above showed significantly greater reduction on the total score (SMD = −1.70, *I*^2^ = 31.2 %, 95% CI −2.04 to −1.36, *p* = 0.03), as compared to those who participated in less than 12 weeks of exercise intervention (SMD = −1.18, *I*^2^ = 0%, 95% CI−1.35 to −1.02, *p* < 0.00001).

**Figure 14:**
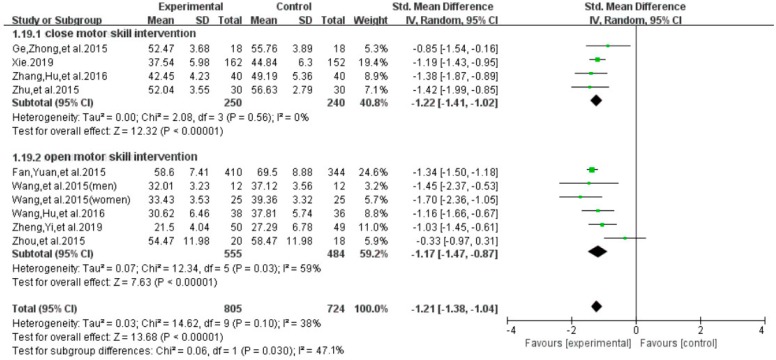
Effect of different exercise interventions on smartphone addiction.

With regard to the exercise interventions, results of subgroup analysis indicated a significant group difference on the total score of smartphone addiction (SMD = −1.21, 95% CI −1.38 to −1.04, *p* = 0.037, Figure 14). Furthermore, results indicated that individuals with a smartphone addiction who participated in exercise of close motor skills showed a significantly greater reduction on the total score (SMD = −1.22, *I*^2^ = 0 %, 95% CI −1.41 to −1.02, *p* = 0.56), as compared to those who participated in exercise intervention of open motor skill (SMD = −1.17, *I*^2^ =44%, 95% CI−1.47 to −0.0.87, *p* = 0.03).

## 4. Discussion

This is the first systematic review with quantitative data analysis on the effect of exercise in individuals with smartphone addiction. We identified nine RCTs (including 1582 participants) that met our inclusion criteria. Results in the current review indicate that exercise has rehabilitative effects on reducing total score of the addiction level and its four subscales (withdrawal symptom, highlight behavior, social comfort, and mood change) among individuals with smartphone addiction. At the same time, based on GRADE quality evaluation, the results were as follows: we are confident that the true effects of exercise on social comfort and mood change are similar to the estimated effects by meta-analyses, and the true effects of exercise on total score, withdrawal symptoms and highlight behavior are most likely close to the estimated effects by meta-analyses. Such positive findings not only support previously published reviews investigating the rehabilitative effects of exercise interventions for individuals with substance use disorder [38,39], but psychological researchers also agree that frequent smartphone use is associated with less physical activity [22,40,41].

The study included participants aged between 18 and 22. According to the World Health Organization, among those addicted to smartphone use, the younger population accounts for a significant proportion [42]. Increased academic pressure, poorly-developed cognitive levels and dissatisfaction with the external environment has led to students often adopt negative ways of using some of the functions in the mobile phone to cope with frustration, e.g., watching videos and playing games for a long time. Some of the features in the phone may induce a transient, positive affective response, but may also induce feelings of hopelessness and depression [1]. Excessive smartphone use may also lead to more impulsive smartphone use, and, subsequently, a decrease in restraint and ability to successfully manage their behaviour, which may lead to further smartphone reliance for college students and further suppression of their control. Decreased control may lead to increases in negative emotional experiences and an increase in negative emotional experience may reduce an individual’s ability to suppress and control undesired behaviors [43]. Relatedly, research has shown that adolescents with greater negative emotions were more likely to suffer negative symptoms of mobile phone use [44]. Similarly, excessive mobile phone use may compromise social relationships. Participating in physical activity may, potentially, counteract this effect by displacing time spent on the phone as well as be engaging social counterparts in the physical activity [45]. Such effects may have a pronounced influence on overall quality of life [46,47].

With regard to the addiction level, we found a large effect size on the total score. It is not surprising that chronic exercise engagement may help to reduce smartphone addiction level, given that both exercise and smartphone use may activate similar neurophysiological pathways in the brain. For example, research demonstrates that addiction to electronic devices affects brain areas associated with reward and loss of impulse control. Regarding the former, addiction to electronic devices is associated with dopamine release in a manner similar to that observed with drug use [48]. Chronic exercise engagement may help to reduce addiction to electronic devices via its own effects on reward stimulation. Research demonstrates that both forced and voluntary exercise increases reward-related neural plasticity in key reward-based brain structures, such as the dorsal striatum, nucleus accumbens, and lateral ventral tegmental area [49]. In addiction exercise-induced alterations in reward-based brain structures, exercise may help to attenuate addictive behaviors via its effects on impulse control. Behavioral addictions, a type of impulse control disorder [50], may be attenuated with chronic exercise engagement given the role of exercise on prefrontal cortex-dependent executive function. In a recent meta-analysis among children and adolescents, chronic exercise engagement improved overall executive function and inhibitory control [51]. From a neurophysiological perspective, exercise has been shown to improve inhibitory control via better performance on allocation of attention, which was well explained by a larger amplitude of the P3 event-related potential [52]. In addition to overall attenuation of addiction via chronic exercise engagement, exercise was also favorably associated with each of the addiction symptoms, such as withdrawal and mood changes. Assuming exercise induces similar reward-based effects when compared to electronic use, it is not surprising that the disuse of electronics attenuates withdrawal symptomology. Further, exercise, even among children and adolescents, has been shown to improve a range of different moods. Thus, collectively, exercise may help to reduce key symptoms of addiction, and ultimately, attenuate addictive behavior.

A notable observation of our meta-analysis was that the duration of chronic exercise played a moderating role in addiction symptomology. Chronic exercise of at least 12 weeks was most favorably associated with less addictive behavior. As previously stated, some of the potential benefits associated with exercise involve adaptations to key brain structures involved in reward and inhibitory control. It is possible that longer exercise programs (e.g., 12+ weeks) are needed for such brain-related morphological adaptations to occur.

Study limitations in this current review need to be pointed out. The GRADE evidence showed that the evidence quality of two outcome indicators was medium, and the quality of three outcome indicators was low. Only one study used follow-up measurements, so positive results regarding the long-term effects of physical activity on this domain still remain elusive. Finally, participants recruited in these studies were from China (mainland), and thus, generalizability is limited to this population.

## 5. Conclusions

In summary, this meta-analysis demonstrates that chronic exercise engagement is associated with less smartphone addiction and fewer withdrawal and mood-related symptoms. Future work should continue to employ randomized controlled designs, but aim to improve their methodology by ensuring allocation concealment, blinding and preventing smartphone use while exercising, when appropriate. In addition, the influence of different exercise on smartphone addiction remains to be further explored, which also provides reference for the improvement of smartphone addiction through exercise in the future.

## Figures and Tables

**Table 1:** Characteristics of included studies.

Reference	Location (Language)	Participant Characteristics			Intervention Program	Motor Skill Training			Outcome Measured	Adverse Event; Follow-Up
		Sample Size	Smart Phone Addiction Level	Mean Age or Age Range		Frequency (weekly)	Time (min)	Duration (week)		
Zhu, et al. (2015) [25]	Zhejiang, China (Chinese)	60	Mild-to-moderate level	20.72 ± 1.30	EG: Taijiquan CG: Usual Care	3	60	8	MPATS (WS; SC; MC; HBsalience)	No No
Zhang, Hu, et al. (2016) [26]	Hunan, China (Chinese)	80	Mild-to-moderate level	18–22	EG: Run CG: Usual Care	2	45	8	MPATS (WS; SC; MC; HBsalience)	No No
Zhou, et al. (2015) [27]	Zhengzhou, China (Chinese)	18	Severe level	20.33 ± 1.64 (freshman)	EG: Bicycle + Basketball + Badminton CG: Usual Care	3	60	12	MPAI (WS, HBuncontrolled)	No No
Wang, et al. (2015) [28]	Taiyuan, China (Chinese)	73	Mild-to-moderate level	18–22	EG: Basketball + Badminton + Football + Tennis CG: Usual Care	3	45	12	MPATS (WS; SC; MC; HBsalience)	No No
Fan, Yuan, et al. (2015) [29]	Nanjing, China (Chinese)	754	NR	18–22 (freshman)	EG: Basketball + Badminton + Football + Tennis CG: Usual Care	5	45–60	12	MPATS (WS; SC; MC; HBsalience)	No No
Wang, Hu, et al. (2016) [30]	Shandong, China (Chinese)	49	Mild-to-moderate level	19.71 ± 1.52	EG: Basketball + Badminton + Dance CG: Usual Care	3	90	10	MPATS (WS; SC; MC; HBsalience)	No No
Ge, Zhong, et al. (2015) [31]	Nanchang, China (Chinese)	36	Severe level	20.13 ± 1.35	EG: Bicycle + Run CG: Usual Care	3	120	10	SAS-C (WS, SC, MC)	No No
Zheng, Yi, et al. (2019) [32]	Hunan, China (Chinese)	99	Mild-to-moderate level	18–20	EG: Basketball + Badminton + Football + Tennis CG: Usual Care	4	60	8	MPAI	No No
Xie (2019) [33]	Shangxi, China (Chinese)	544	Severe level	18–22	EG: Baduanjin CG: Usual Care	4	40–60	8/16/24	MPAI (WS, HBuncontrolled)	No Yes

Note: EG = experimental group; CG = control group; WS = withdrawal symptoms; HB = highlight behavior; SC = social comfort; MC = mood change; NE = negative effect; MPATS = smartphone addiction tendency scale; SAS-C = smart phone addiction scale for college students; MPAI = smartphone addiction index; NR = not reported. Smart phone addiction level: 40 to 60 points = mild-to-moderate level; more than 60 points = severe level. Highlight behavior: uncontrolled and salience are emphasizing the importance of using mobile phone on individuals, so we merged them into one item of highlight behavior.

**Table 2:** Study quality assessment.

Author [Reference]	Item 1	Item 2	Item 3	Item 4	Item 5	Item 6	Item 7	Item 8	Item 9	Score
Zhu, et al. (2015) [25]	1	1	0	1	0	1	1	1	1	7
Zhang, Hu, et al. (2016) [26]	1	1	0	1	0	1	1	1	1	7
Zhou, et al. (2015) [27]	1	1	0	1	0	1	1	1	1	7
Wang, et al. (2015) [28]	1	1	0	1	0	1	1	1	1	7
Fan, Yuan, et al. (2015) [29]	1	1	0	1	0	1	1	1	1	7
Wang, Hu, et al. (2016) [30]	1	1	0	1	1	1	1	1	1	8
Ge, Zhong, et al. (2015) [31]	1	1	0	1	1	1	1	1	1	8
Zheng, Yi, et al. (2019) [32]	1	1	0	1	0	1	1	1	0	6
Xie (2019) [33]	1	1	0	1	0	1	1	1	1	7

Note: Item 1 = eligibility criteria; Item 2 = randomization; Item 3 = concealed allocation; Item 4 = similar baseline; Item 5 = blinding of assessors; Item 6 = outcome measures integrity; Item 7 = attrition rate of more than 85%; Item 8 = between-group comparison; Item 9 = point measure and measures of variability (value 1 means that the corresponding item was explicitly described and present in details; 0 means that the corresponding item was absent, inadequately described, or unclear).

**Table 3:** Synthesized results for the effects of exercises versus control condition.

Outcomes	Pairwise Comparison	Sample Size		Meta-Analysis			Heterogeneity	
		Motor Skill Group	Control Group	SMD	95% CI	*p*-Value	*I*^2^%	df (Q)
Total	10	792	790	−1.30	−1.53 to −1.07	0.00	62%	9
WS	9	755	741	−1.40	−1.73 to −1.07	0.00	81%	8
HB	8	735	724	−1.95	−2.30 to −1.59	0.00	79%	7
SC	7	583	581	−0.99	−1.18 to −0.81	0.00	21%	6
MC	6	556	554	−0.50	−0.69 to −0.31	0.00	25%	5

Note: WS = withdrawal symptoms; HB = highlight behavior; SC = social comfort; MC = mood change.

**Table 4:** GRADE assessment of evidence certainty for exercise effects.

Outcomes	Presence of Downgrading Item of GRADE					Level of Certainty of Evidence
	Publication Bias	Inconsistency	Indirectness	Imprecision	Risk of Bias	
Total	No	No	No	Yes	Yes	III (Low)
WS	No	No	No	Yes	Yes	III (Low)
HB	No	No	No	Yes	Yes	III (Low)
SC	No	No	No	No	Yes	II (Moderate)
MC	No	No	No	No	Yes	II (Moderate)
